# PPAR-Responsive Elements Enriched with Alu Repeats May Contribute to Distinctive PPARγ–DNMT1 Interactions in the Genome

**DOI:** 10.3390/cancers13163993

**Published:** 2021-08-07

**Authors:** Amit Sharma, Fabian Tobar-Tosse, Tikam Chand Dakal, Hongde Liu, Arijit Biswas, Athira Menon, Anoosha Paruchuri, Panagiotis Katsonis, Olivier Lichtarge, M. Michael Gromiha, Michael Ludwig, Ingo G. H. Schmidt-Wolf, Frank G. Holz, Karin U. Loeffler, Martina C. Herwig-Carl

**Affiliations:** 1 Department of Ophthalmology, University Hospital Bonn, 53127 Bonn, Germany; Frank.Holz@ukbonn.de (F.G.H.); Karinloeffler@uni-bonn.de (K.U.L.); Martina.Herwig@ukbonn.de (M.C.H.-C.); 2 Department of Neurosurgery, University of Bonn, 53127 Bonn, Germany; 3 Department of Basic Sciences for Health, Pontificia Universidad Javeriana Cali, Cali 760031, Colombia; ftobar@javerianacali.edu.co; 4 Department of Biotechnology, Mohanlal Sukhadia University Udaipur, Udaipur 313001, India; tc.dakal@mlsu.ac.in (T.C.D.); aathimenon143@gmail.com (A.M.); 5 State Key Laboratory of Bioelectronics, Southeast University, Nanjing 210096, China; liuhongde@seu.edu.cn; 6 Institute of Experimental Hematology and Transfusion Medicine, University Hospital Bonn, 53127 Bonn, Germany; arijit.biswas@ukbonn.de; 7 Department of Internal Medicine, Division of Medical Oncology and Comprehensive Cancer Center, The Ohio State University, Columbus, OH 43210, USA; Anoosha.Paruchuri@osumc.edu; 8 Computational and Integrative Biomedical Research Center, Baylor College of Medicine, Houston, TX 77030, USA; katsonis@bcm.edu (P.K.); lichtarg@bcm.edu (O.L.); 9 Department of Biotechnology, Bhupat and Jyoti Mehta School of BioSciences, Indian Institute of Technology Madras, Chennai 600036, India; gromiha@iitm.ac.in; 10 Department of Clinical Chemistry and Clinical Pharmacology, University Hospital Bonn, 53127 Bonn, Germany; mludwig@uni-bonn.de; 11 Department of Integrated Oncology, CIO Bonn, University Hospital Bonn, 53127 Bonn, Germany; Ingo.Schmidt-Wolf@ukbonn.de

**Keywords:** cancer, repetitive sequences, evolutionary action score, uveal melanoma, Alu repeats, PPARγ, DNMT1

## Abstract

**Simple Summary:**

This study aimed to explore the potential role of PPARγ–DNMT1 interaction through PPAR-responsive elements (PPREs), which we have found to be enriched with Alu repeats. Apart from protein–protein interactions and co-expression in multiple cancer types, we exclusively described a prognostic role for PPARγ in uveal melanoma (UM).

**Abstract:**

Background: PPARγ (peroxisome proliferator-activated receptor gamma) is involved in the pathology of numerous diseases, including UM and other types of cancer. Emerging evidence suggests that an interaction between PPARγ and DNMTs (DNA methyltransferase) plays a role in cancer that is yet to be defined. Methods: The configuration of the repeating elements was performed with CAP3 and MAFFT, and the structural modelling was conducted with HDOCK. An evolutionary action scores algorithm was used to identify oncogenic variants. A systematic bioinformatic appraisal of PPARγ and DNMT1 was performed across 29 tumor types and UM available in The Cancer Genome Atlas (TCGA). Results: PPAR-responsive elements (PPREs) enriched with Alu repeats are associated with different genomic regions, particularly the promotor region of DNMT1. PPARγ–DNMT1 co-expression is significantly associated with several cancers. C-terminals of PPARγ and DNMT1 appear to be the potential protein–protein interaction sites where disease-specific mutations may directly impair the respective protein functions. Furthermore, PPARγ expression could be identified as an additional prognostic marker for UM. Conclusions: We hypothesize that the function of PPARγ requires an additional contribution of Alu repeats which may directly influence the DNMT1 network. Regarding UM, PPARγ appears to be an additional discriminatory prognostic marker, in particular in disomy 3 tumors.

## 1. Introduction

Studies from the cancer genome suggest that cancer is not merely restricted to certain mutations in genes (oncogenes, tumor suppressor genes, driver genes). Instead, a crosstalk between genetic factors and epigenetic modulators orchestrates the heterogeneous and inter-individual disease pattern [1,2,3]. In particular, genes with a broader mechanistic involvement in several diseases such as PPARγ (peroxisome proliferator-activated receptor gamma), which is mainly involved in the regulation of metabolic processes, but is also mutated or overexpressed in several human cancers, should be more deeply investigated for epigenetic relations. Previously, we have shown that PPARγ is expressed in uveal melanoma (UM) which is the most frequent intraocular primary tumor in Caucasian adults [4]. PPARγ is involved in tumorigenesis since it is expressed in different tumors including their microenvironment and promote polarization of anti-inflammatory pro-tumorigenic M2 macrophages [5,6,7,8]. However, it is still under debate under which conditions pro- or anti-carcinogenic effects dominate (reviewed in [9]).

In recent years, there has been a growing debate about the common biological mechanisms in metabolic diseases and cancer [10,11,12], and genes such as PPARγ aroused major interest. Peroxisome proliferator-activated receptors (PPARs) are well known to have key functions in many physiological processes such as lipid metabolism, cell growth, differentiation, and apoptosis. Among the PPAR genes (PPARα, PPARβ/δ, PPARγ), PPARγ is strongly expressed in adipose tissue (brown and white) and weakly expressed in intestinal epithelium, retina, immunological systems (including macrophages) and skeletal muscle [13,14]. Regarding transcriptional regulation, it is well established that ligand-activated PPARγ forms a heterodimer with the retinoid X receptor (RXR) and then binds to specific DNA sequences (PPAR-responsive elements, also called PPREs) in the promoter region of target genes. Fang et al. developed a machine learning method and predicted novel PPAR target genes (PPARα: 83 targets, PPARβ/δ: 83 targets, PPARγ: 104 targets) using different tissue types and species [15]. Emerging evidence suggests that PPARγ and DNA methyltransferase 1 (DNMT1) molecular pathways are intertwined [16,17]. DNMT1 is necessary to maintain methylation in the genome and has also been linked to several human diseases, ranging from cancer to neurological abnormalities [18,19]. Moreover, DNMT1 has been shown to promote activation of macrophage M1 by suppressing KLF4 expression in atherosclerosis [20].

Herein, we investigated a yet to be defined role of the PPARγ–DNMT1 interaction through PPREs, which we have found to be enriched with Alu repeats in this particular genomic region. Apart from protein–protein interactions and co-expression in multiple cancer types, we exclusively described a prognostic role for PPARγ in UM.

## 2. Materials and Methods

### 2.1. Identification and Configuration of Repetitive Elements

We explored a 4kb and a 2kb region, respectively, upstream of the transcription start sites (TSS) of the DNMT1 gene for the identification of PPREs to identify possible configurations of Alu repetitive elements. The sequences from the Alu family were retrieved from the UCSC genome browser by defining the genome (assembly GRChg38) as data source. The Alu consensus sequence to describe the significance of candidate motifs was optimized using two steps: 1. by scoring the redundancy using the assembly program CAP3 (http://seq.cs.iastate.edu/cap3.html, accessed on 29 November 2019) to minimise the number of identical sequences or copies, 2. by multiple alignments of the resulting sequences in each subfamily to extract the consensus sequence using the program MAFFT (https://mafft.cbrc.jp/alignment/software/, accessed on 4 December 2019). The final Alu map was defined by multiple alignments of all consensus sequences in the subfamily and is represented in a sequence logo plot. The phylogenetic analysis was performed to assign the motif to the closest Alu subfamily, and the phylogenetic tree was created using Neighbor Joining method and MEGA software; Version 10.1.8 https://www.megasoftware.net/ (accessed on 27 June 2020). For further analysis, we named this Alu embedded PPRE sequence as PPREs-Alu motif and extended the protein–protein association analysis by adding DNMT1 interactors and UM-specific driver genes using string tool (https://string-db.org/, accessed on 28 January 2020). The regulatory network based on the density and profile of the transcription factors was modelled by TRANSFAC and JASPAR using PWMs in the web-based enrichment analysis tool Enrichr [21].

### 2.2. Structural Modelling of PPAR-Responsive Element in DNMT1 Promoter Region

The 3D structural model of the PPARγ responsive element present in the DNMT1 promoter region was generated using default parameter of the webserver SFC Bio (http://www.scfbio-iitd.res.in/, accessed on 6 November 2019) with the nucleotide sequence of the responsive element. Subsequently, the PDB (Protein Data Bank) structure of the receptor PPARγ was also retrieved from RCSB protein database with the PDB-ID: 6FZP and both PDB files were subjected to template-free protein-DNA docking using HDOCK (http://hdock.phys.hust.edu.cn/, accessed on 13 March 2019) [22]. Briefly, the PDB file of the protein was uploaded to the input receiver molecule and the response element was fed to the input ligand molecule. The docking was performed template-free, and no specific residues were set. The models were submitted under the job ID: 5e6c7d167c802. The top scoring docking pose was selected for the analysis of the DNA-protein interaction. Further analysis was done using UCSF Chimera 1.14. The interacting partners of PPARγ–DNMT1 that show spatial and genetic interaction and the ones that co-express/co-locate were identified by Genemania (https://genemania.org/, accessed on 6 November 2019) using the default setting [23].

### 2.3. PPARγ and DNMT1 Gene Expression and Mutational Spectrum

To obtain an overview in cancer landscape, the gene expression data for PPARγ and DNMT1 were retrieved from the TCGA project and a total of 9523 samples across 29 tumor types were downloaded, including 8811 tumor tissues and 712 non-tumor (control) tissues. The Pearson correlation coefficient (r) of the expression between PPARγ and DNMT1 was calculated in cancer and control samples, respectively. The expression level is represented as FPKM (fragments per kilobase of exon model per million reads mapped) value. In addition, the mutational spectrum of PPARγ–DNMT1 was derived from 28 cancer types using the Catalogue of Somatic Mutations in Cancer (COSMIC) database [24]. This unique set of data (PPARγ: 107 mutations; DNMT1: 453 mutations) across cancer types, out of which the majority (90%) are missense mutations, was further used to determine the pathogenic probability through evolutionary action (EA) scores [25]. The distribution of EA scores was compared to the random nucleotide changes in each gene, using Kolmogorov–Smirnov test as described elsewhere [26].

### 2.4. Analysis of Putative PPARγ–DNMT1 Protein–Protein Interactions

Since no data on PPARγ–DNMT1 protein–protein interactions were available, we first searched the STRING network database (https://string-db.org/, accessed on 20 February 2020) using the Uniprot entries of PPARγ and DNMT1 as default inputs [27]. In addition, we also used the amino acid sequence from these Uniprot entries in the protein–protein residue interaction prediction server BIPSPI (http://bipspi.cnb.csic.es/xgbPredApp/, accessed on 1 March 2020; sequence information interface) [28]. Briefly, this server uses a machine learning-based method for the prediction of partner-specific protein–protein interaction sites. To obtain a structural perspective, we also downloaded the PDB files corresponding to PPARγ and DNMT1 proteins (PDB file: 4WXX; resolution 2.62 Å and 3DZY; resolution 3.10 Å) and submitted them to the HDOCK protein docking server under default conditions. The highest scoring docking poses were evaluated with respect to the reported mutational distribution.

### 2.5. Analysis of PPARγ–DNMT1 in Uveal Melanoma

For UM, the data of 80 cases from the TCGA project were retrieved and based on the chromosome 3 status it was further divided into two groups: disomy 3 (49 cases) and monosomy 3 (31 cases) of chromosome 3. The Kaplan–Meier survival model was used to estimate the survival difference

## 3. Results

### 3.1. Distinctive Genomic Architecture of PPREs-Alu Motifs in the Promoter of DNMT1

**Figure 1:**
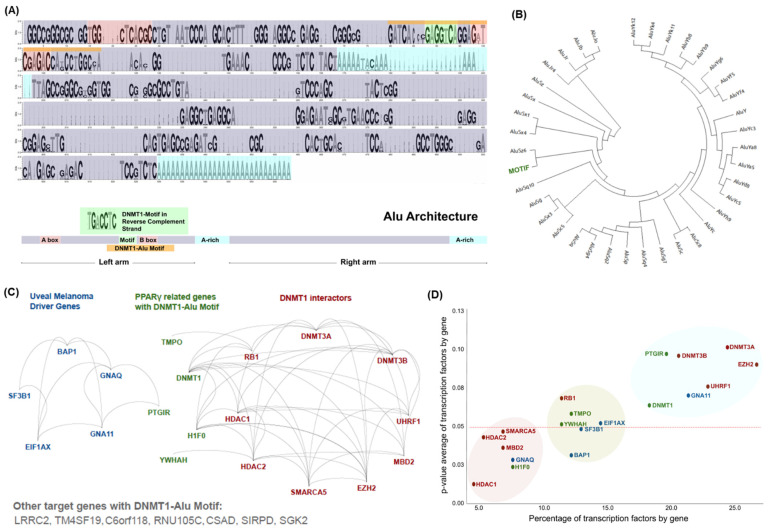
PPREs and Enrichment of Alu repeats. (**A**) The consensus sequences of Alu repeats present in the PPREs. (**B**) 37 Alu subfamilies and significant association with AluJ, AluS, and AluY members in the consensus sequence. (**C**) The Alu-enriched motif structure is present in a few other genes mainly in the proximity to the DNMT1 network and associated with UM-specific driver genes. (**D**) Transcription factors related to these genes marked by significant p-value association.

We first investigated the promoter region of DNMT1 in the range of 4kb upstream of the transcriptional start sites (TSS) to define potential PPREs. The analysis revealed a PPRE and two proximal Alu repeats configured with low complexity and L1 repeats (Alu-Alu-Low complexity-Line/L1) in the vicinity of the identified PPRE. We further defined this PPREs-Alu repeat configuration in the promoter of all coding genes primarily to examine the significance of the Alu family in these PPREs (Figure 1A). The analysis showed a conserved sequence of Alu family repeats in the consensus sequences of the 37 Alu subfamilies. Importantly, we identified the PPARγ binding site (TGACCTC) in the left arm of the Alu structure located specifically next to the B-box which is a conserved site of the promoter of Pol III. As this motif is strongly conserved in the reverse complement strand, we investigated the distribution of this DNMT1 motif in the phylogenetic hierarchy of the Alu family. Regarding the grade of conservation, we observed that Alus within the DNMT1 motif belong to the subfamilies AluJ (the oldest with deleterious sequences) and AluS (the second oldest and active element) (Figure 1B). For further analysis we named this as PPREs-Alu motif. To investigate similar motifs in the genome, we narrowed the 2kb upstream of the TSS region (Alu conserved site) and identified 11 genes with a similar Alu configuration in their promoters (Figure 1C). Importantly, three genes among them were present as functional associations with two categories: UM (PTGIR gene) and DNMT1 (TMPO, histone H1F0, YWHAH). Although we cannot determine any direct functional associations between these genes, we present the first evidence of a possible co-regulatory network based on PPREs-Alu motifs. The transcription factor (TF) density of these genes identified 132 TF differentiating these genes with significant p-value association (Figure 1D). Subsequently, these genes with a *p*-value < 0.05 may be most affected in a PPARγ knockout condition with the H1F0 gene (H1 histone family member 0) showing a severe phenotypic impact. We also found a significant p-value for BAP1, indicating that its TFs are highly related and may be sensitive to PPARγ knockout. Experimental evidence will be needed to investigate these hypotheses.

### 3.2. PPREs and DNMT1 Interactions

**Figure 2:**
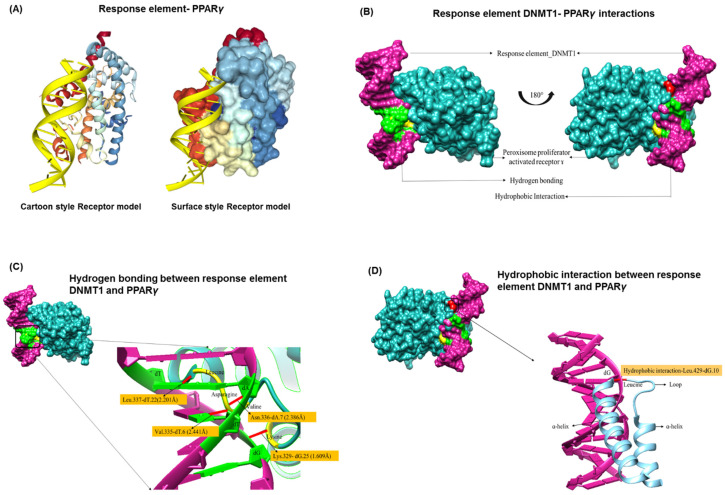
Protein-DNA interactions between PPARγ and DNMT1 via PPREs. (**A**,**B**) The highly significant protein-DNA interaction model with specific residues (presented in cartoon style and surface style) interacting with the response element. (**C**) The interaction through the hydrogen bond between dG-25 and Lys at 329th position; dT-6 and Val at 335th position; dA-7 and Asn at 336th position; dT-22 and Leu at 337th position. (**D**) The hydrophobic interaction between dG-10 and Leu residue at 429th position is marked.

To define the binding of PPREs-Alu motifs in DNMT1, we generated 3D structure of PPREs from their nucleotides and performed docking studies with DNMT1. The top ten model predictions made by HDOCK (with receptor and PDB-ID: 3DZY) showed a complete match of 95.2% with the receptor sequence and 83.3% for the ligand molecule with an average docking score of −250. In addition, an energy score for docking and a RMSD value of 50 Å was obtained. The analysis revealed a highly significant protein-DNA interaction model with specific residues (represented in cartoon style and surface style) interacting with the response element (Figure 2A,B). The interaction through the hydrogen bond was found between dG-25 and Lys at 329th position; dT-6 and Val at 335th position; dA-7 and Asn at 336th position; dT-22 and Leu at 337th position (Figure 2C). In addition, the hydrophobic interaction between dG-10 and Leu residue at 429th position is shown in Figure 2D. Since, all the interactions (both hydrogen and hydrophobic) occur in the ligand binding domain (LBD) region, any mutation or deletion in this region will result in the loss of ligand recognition activity of PPARγ. The analysis also showed that all interacting residues belong to the LBD of PPARγ (position: 238–503) which plays a crucial role in the activity of the ligand-mediated nuclear receptor (NR) and in ligand recognition. The interacting partners of PPARγ–DNMT1 which show spatial and genetic interaction, were also investigated (Figure S1). The genetic interactions of DNMT1 to infer protein–protein interactions and co-functionality showed 20 related genes with a total of 287 links, while the spatial interaction showed 19 related genes and 15 genes co-expressed with DNMT1. On the other hand, the PPARγ gene was connected to 20 related genes via a total of 98 connections with spatial interactions with 7 genes and co-expression with 14 genes. When both sets of genes were analyzed for mutual interactions, some common interaction partners were found, namely NCOA6, HDAC1, RB1. Among them, NCOA6 (Nuclear Receptor Co-activator 6) is a multifunctional co-regulator or co-activator necessary for the transcriptional activation of a wide range of target genes [29]. The histone deacetylase HDAC1 physically interacts and cooperates with RB1 (RB, retinoblastoma associated protein) via the LXCXE motif and this Rb/HDAC1 complex plays a key role in controlling cell proliferation and differentiation [30]. Taken together, the co-expression of these genes (NCOA6, HDAC1, RB1) could play a role in various types of cancer.

### 3.3. PPARγ–DNMT1 Cancer Landscape and Protein–Protein Interactions

**Figure 3:**
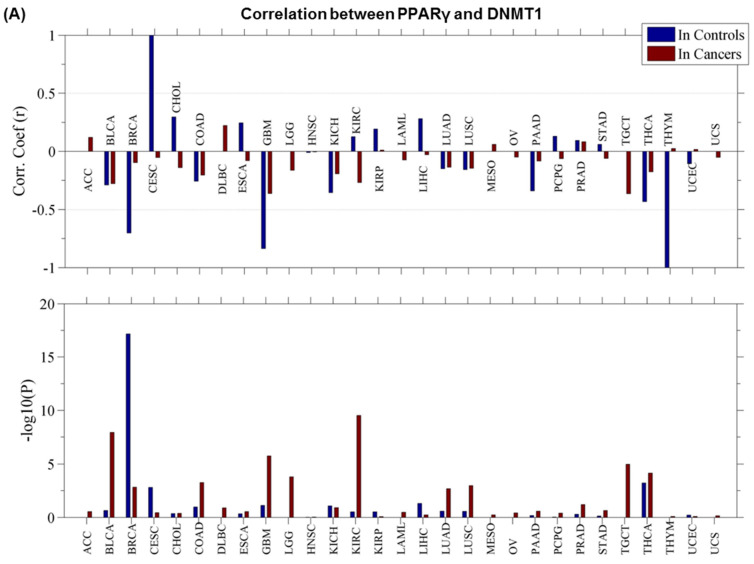

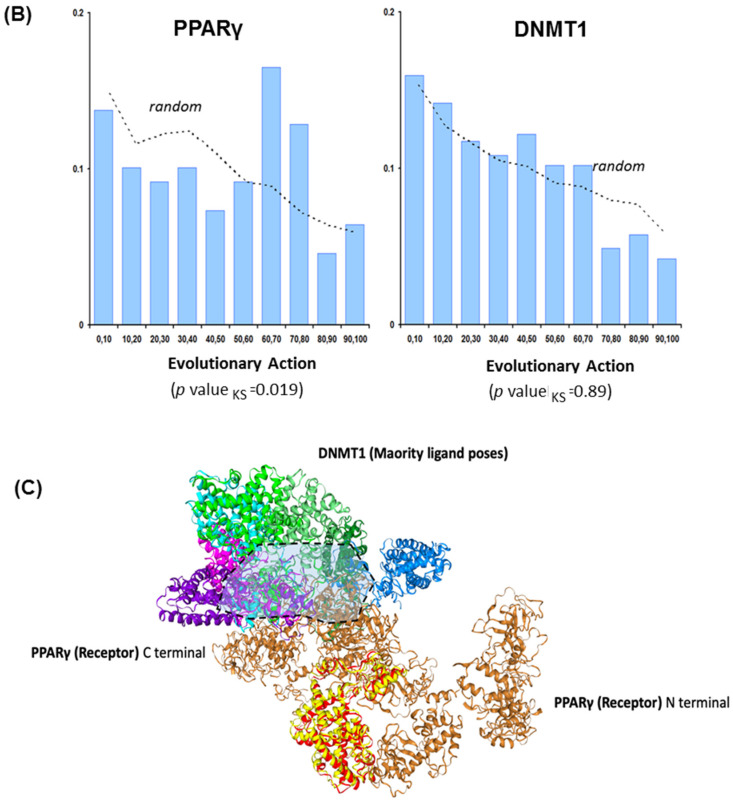
PPARγ–DNMT1 protein–protein interactions and cancer landscape. (**A**) The Pearson correlation coefficient (r) of expression between PPARγ and DNMT1. Top panel, the coefficients in control samples and cancer samples, respectively; Bottom panel, the significance (*p*-value (-log10)) of each coefficient by *t*-test: (**B**) Evolutionary action (EA) scores showing significance levels. (**C**) High scoring docking poses from the docking simulation for PPARγ–DNMT1 proteins. In the docking simulation PPARγ (camel colored) is shown as receptor, DNMT1 (several colors for different docking positions) as ligand and protein backbone in ribbon format. The highly probable interface is depicted with a shaded region.

The analysis of the expression pattern of PPARγ and DNMT1 in different types of cancer (using TCGA) revealed that both genes are dysregulated (Figure 3A). The Pearson correlation coefficient (r) of expression between these genes showed strong coefficients in controls (compared to cancers) in the case of CESC, BRCA, GBM, and THYM. However, a very weak correlation (between −0.5 and 0.5) was observed in COAD, ESCA, KICH, LUAD, LUSC, PAAD, STAD, THCA, and UCEC. This suggests that the expression pattern of both genes is disturbed in cancer. Moreover, both PPARγ and DNMT1 are reported to be enriched with mutations in various cancer types (*n* = 28; Figure S3). The evolutionary action (EA) score for these mutations showed that PPARγ variants are more likely to be of an oncogenic type (EA score: 60–80, *p*-value KS = 0.019) compared to the DNMT1 variants (*p*-value KS = 0.89) (Figure 3B). Notably for DNMT1, the very small increase in EA intermediates may be due to the cancer types included in the analysis for which DNMT1 is not a driving gene.

Due to the lack of protein–protein interaction data in the literature, we evaluated PPARγ–DNMT1 proteins in the STRING network database which did not show strong experimental support for the actual protein–protein interaction. However, there was limited experimental evidence based on co-immunoprecipitation data that suggested an interaction between putative homologs of these two proteins in rat models (Figure S2) [31]. Importantly, the prediction of residue interactions indicated that it is mainly the residues at the C-terminus of both proteins that are part of the protein–protein interaction interface (Figure 3C). The results were also supported by the structural docking of these two proteins as the majority of high score docking poses were clustered around the C-terminal of these two proteins. The mutations occurring at the C-terminus of PPARγ–DNMT1 can interrupt protein–protein interactions and may impact the disease phenotype. Given that PPARγ–DNMT1 homologous proteins interact in the rat, it is likely that they could interact in humans as well, so further studies in these areas are warranted.

### 3.4. PPARγ–DNMT1: Insights from Uveal Melanoma

**Figure 4:**
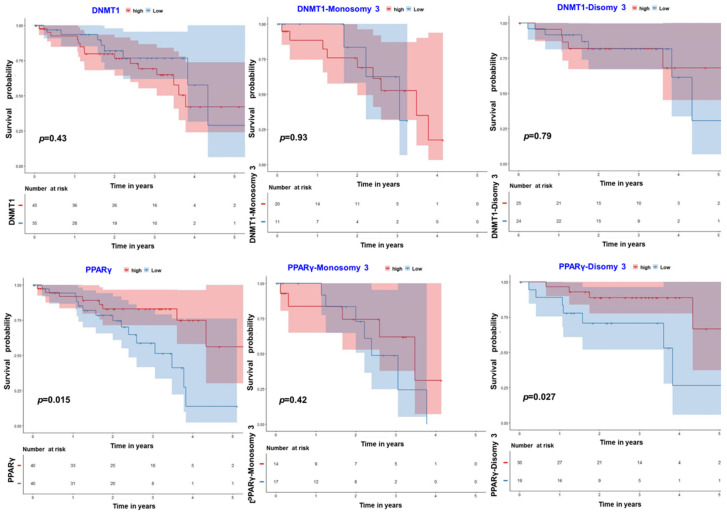
Prognostic analysis of PPARγ–DNMT1 in the TCGA UM dataset. Classification of 80 cases in TCGA database of UM. The prognostic values of the PPARγ–DNMT1 genes were analyzed for all cases as well as monosomy 3 and disomy 3 tumors. Overall survival curves were plotted for the cases with high and low expression of the two genes with Kaplan–Meier survival model. Left panels, all cases; middle panels, monosomy 3 class; right panels, disomy 3 class.

80 UM cases from the TCGA database were investigated and a survival analysis comparing two main groups was performed. Of the 80 UM cases in this database 31 cases were from monosomy 3 tumors (with a high likelihood for metastasic disease) and 49 from disomy 3 tumors (with a low likelihood for metastastic disease) (Figure 4). We did not observe a survival difference between the cases associated with high and low expression of DMNT1 (*p*-value = 0.43) (Figure 4, upper panel). For PPARγ a survival difference was found for the entire cohort (*p*-value = 0.015) independent of the chromosome 3 status. For patients with disomy 3 tumors (*p*-value = 0.027) but not for monosomy 3 tumors (*p*-value = 0.42) PPARγ expression was an additional predictor of survival (Figure 4, lower panel) with a high PPARγ expression being associated with a longer survival time. An additional analysis of the disomy 3 tumors revealed that a low expression of PPARγ was not related to a SF3B1 mutation as SF3B1 mutations were mainly detected in UM with a high PPARγ expression (*n* = 17) and only in one patient with a low PPARγ expression (*n* = 1).

## 4. Discussion

It is well known that the activities of PPARs and DNMTs are pivotal, and their dysregulation is associated with diseases such as different types of cancer. Pazienza et al. evaluated the expression of PPARγ, DNMT1, and DNMT3B and their correlation with clinical-pathological features in patients with pancreatic cancer [16]. Recently, a study demonstrated that the activated PPARγ physically interacted with DNMT1 and HDAC1 in a CpG island on the Hic-1 gene to assemble PPARγ/DNMT1 and PPARγ/HDAC1 protein complexes in hepatocarcinoma cells [32]. It was also reported that PPAR-γ is a target of DNMT1-regulated DNA methylation and is involved in DNMT1-mediated chronic inflammation and atherosclerosis development [17]. Considering the above-mentioned studies and the important role of DNMTs in cancer, we investigated the promoter region of DNMT1 in the range of 4kb upstream of the transcriptional start sites (TSS) and identified potential PPREs with two proximal Alu repeats in the vicinity (known as PPREs-Alu motif). Our comprehensive analysis identified 11 genes with a similar configuration that were distinguished by distinct transcription factors. To define the binding of PPREs-Alu motifs in DNMT1, we generated the 3D structure of PPREs from their nucleotides and performed docking studies with DNMT1 that revealed a highly significant protein-DNA interaction. It was also found that the interaction partners of PPARγ–DNMT1 have spatial and genetic interactions. Moreover, the expression of PPARγ and DNMT1 was found to be dysregulated in various cancers. The evolutionary action score showed that PPARγ variants were more likely to be oncogenic in nature compared to the DNMT1 variants. Our analysis also revealed that the mutations occurring at the C-terminus of PPARγ–DNMT1 can interrupt their protein–protein interactions and may impact the disease phenotype.

We have previously reported that PPARγ is expressed in UM, which is the most frequent primary intraocular tumor in Caucasian adults with abnormal genetic-epigenetic characteristics and metastatic potential [26,33,34]. Recently, we also reported that DNMT1, along with a few other chromatin-associated genes, is a potential miRNA target cluster embedded within one of the driver genes (BAP1) of UM [26]. In the present study, we found a survival difference in UM of the TCGA database for PPARγ but not for DNMT1. Several chromosomal abnormalities and somatic mutations determine the metastatic risk in UM. The most important factor is the chromosome 3 status with monosomy of chromosome 3 being highly associated with metastatic disease. Other chromosomal aberrations which have been identified as prognostically relevant are on chromosome 1, 6 and 8 [35,36]. Furthermore, five driver genes have been reported with GNA11 and GNAQ mutations representing initiating mutations and BAP1, EIF1AX, and SF3B1 occurring later during the course of the disease. Although BAP1–located on chromosome 3–correlates well with the chromosome 3 status and a BAP1 mutation is associated with a high likelihood for metastatic disease, EIF1AX and SF3B1 occur typically in disomy 3 tumors. EIF1AX mutated tumors usually do not metastasize while SF3B1 is associated with late metastases of disomy 3 tumors [33,37,38,39]. Based on our transcription factor analysis mentioned above, we hypothesize that PPARγ knockout may have a significant effect on BAP1. However, experimental evidence is warranted.

In this study, we showed that PPARγ may serve as an additional prognostic factor in UM patients in general and in UM patients with disomy 3 tumors (but not monosomy 3 tumors) in particular. However, a low and prognostically unfavorite PPARγ expression in disomy 3 tumors was not related to SF3B1 mutations as could have been expected due to its association with late metastasis. Since in a clinical trial (phase I) with a PETT schedule including pioglitazone (a PPARγ agonist) revealed a relatively long survival in two UM patients with liver metastases [40], this is another argument to study the role of PPARγ in UM in more detail.

Taken together, we showed that PPREs-Alu region has the tendency to interact with the DNMT1 protein, which was confirmed by our protein-DNA interaction model (Figure 2). Direct interactions of DNMT1 and PPARy proteins may also occur, as shown in the protein–protein interaction model (Figure 3). We could show DNMT1-PPARY-Alu repeats exhibit potential interdependence at both DNA and protein levels. On a broader perspective, our study raises two important questions, (1) exactly what difference does this PPREs-Alu repeat configuration make in the genome compared to common PPREs, (2) if the involvement of repetitive Alu genomes mediates and extends the function of these pleiotropic PPARγ and DNMT1 genes towards other dysregulated scenarios (e.g., connecting metabolism and cancer). Therefore, further validation with in vivo/in vitro model systems may help to gain better insights into their functional role.

## 5. Conclusions

PPREs enriched with Alu repeats represent discriminative genomic regions and DNMT1 appears to be the main target in this DNA-protein interaction. The structural docking and interface residue interaction predicts the C-terminals of both proteins to be the potential protein–protein interaction sites where disease-specific mutations may directly impair the respective protein functions. PPARγ–DNMT1 co-expression is associated with several cancers while the evolutionary action (EA) score revealed PPARγ variants to be more of an oncogenic type. In the context of UM, PPARγ appears to be an additional discriminatory prognostic marker, in particular in disomy 3 tumors. Considering the pleiotropic nature of PPARγ and DNMT1 there is a need to investigate if their interaction also plays a role in the association between metabolism and cancer.

## Data Availability

Data are contained within the article or Supplementary Material.

## Supplementary Materials

The following are available online at https://www.mdpi.com/article/10.3390/cancers13163993/s1. Supplementary file: Genomic sequence containing two Alus and the response elements. Genomic sequences upstream of DNMT1 where Alu repeats and the PPREs (yellow) were identified, are shown. Figure S1: PPARγ-DNMT1 spatial and genetic interaction. The interacting partners of PPARγ-DNMT1 exhibiting spatial and genetic interaction (in red) and those that are co-expressed and/or co-localized (in light blue) are shown. Figure S2: PPARγ-DNMT1 mutation frequency. The frequency of mutations associated with PPARγ-DNMT1 in cancers (COSMIC data). Figure S3: PPARγ- DNMT1 STRING analysis. (A) PPARγ-DNMT1 proteins from the STRING network database. (B) PPARγ-DNMT1 proteins for interface residue interaction prediction.

## Abbreviations

BRCA: Breast invasive carcinoma, CESC: Cervical squamous cell carcinoma and endocervical adenocarcinoma, COAD: Colon adenocarcinoma, ESCA: Esophageal carcinoma, GBM: Glioblastoma multiforme, KICH: Kidney Chromophobe, LUAD: Lung adenocarcinoma, LUSC: Lung squamous cell carcinoma, PAAD: Pancreatic adenocarcinoma, STAD: Stomach denocarcinoma, THYM: Thymoma, THCA: Thyroid carcinoma, UCEC: Uterine Corpus Endometrial Carcinoma.
