# Supplementary material for: PPAR-Responsive Elements Enriched with Alu Repeats May Contribute to Distinctive PPARγ–DNMT1 Interactions in the Genome
Source: Cancers (Basel). 2021 Aug 7;13(16):3993. doi: 10.3390/cancers13163993 (PMC8391462; doi:10.3390/cancers13163993)
Supplement: Supplementary file 1 [file cancers-13-03993-s001.zip › Supplementary file.pdf]

**Supplementary file: Genomic sequence containing two Alus and the response elements**

chr19:10,197,601-10,201,814

CACCACTGCACTCCATCCAGCCTGGGCAACAGAGCGAGACTCCGTCTCAA
AAACAAACAAAAAGAGCAAATGATTTGAATACATACTTTCCCAAAGGAGA
TATGCAAATAGAAAACAGGCATATGAAAAGATGCTCAACATCACTGGTCA
TCAGGGAAATGCAAATCAAAACCACAATGAAATATCCCTAAGACCCCTTA
GAATGGCCACTGTCAAATAATAAGTGTTGGTGAAGATGTGGAGAAACTGG
AACCCTTGTGCACGGTTGAGAGTCGAATAAAATGGTGGAGCTGCTATGAG
AAACAGCATGGAAGTTCCTCAAAAAAATTAAAAATAGAATTATCATATGA
TCCAGCAATCCCATTTCTGGATATAAATTCAAAAGAATTGAAAAGAGGAT
CTTAAAGAGATATTTGCATACCCGTCTTCATTGCAGCATTAGTCACAACA
GCCAAGAGACAGGAAAAAAAAAAAAAAAAGATCATATTGCTGCTAAGAGAT
GGGGTCTTGCTGTGTTGCCCAGGCAGGTGTCAAACTCCTGGGCTCAAGGC
ATACTCCTACCTCAGCTTCTTAAGTAGCTGGGACTACAGGTATGTGCCAC
CACAATGGGCTAACTTTTTATATTTTTTGTAGAGACAGGATTTCTCCATG
TTGCCCAGGCTGGTCTCAAACTCCTGAGCTCAAGCGATCTGCTGGCCTTG
GCCTCCCAAAGCGCTGGGATTACAGGTGTAAGCCACTGCACCCAGCCTAA
TTCAGTCTTTAAAAAAGAAAAAATGGCCAGGTGCAGTGGCTCACACCTGT
AATCCCAACACTTTGGGAAGCCAAGGCGGGTGGATCACCTGAGGTCAGGA
GTTCGAGACCAGCCTGGCCAACATGGAGAAACCCCATCTCTACTAAAAAT
ACAAAATTAGCTGGACATAGTGGCTCATGCCTATAACCCCAGCTACTCCG
GAGGCTGAGGCAGGAGAATTGCTTGAACCCTGGAGGTGGAGGTTGAGGTA
AGCTGAGATTACACCATTGTACTCCAGCCTGGGCAACAAGAGGGAAACTC
CATCTCAAAATAAATAAATAAATAAATAAATAAATAATATAAAAGTAAAA
ATACAAAATTAGCTGGGTATGGTGGCATGCACCTGTAATCCCAGCTACTT
AGGAGGTTAAGGCAAGAGTACTGCTTGAACCCGAGAGGTGGAGGTTGCAG
TGAGCTGAGATCACACCATTGCACTCCAGCCTGGGTGACAAGAGTGAAAC
TCCGTCTCCAAAAAACAAAAGAAAAGAAAAAATATCTTATTATATGCTAC
AACATGGATGAACCTTGAGGACATTACGCACAGTGAGAGAACCTAATCAC
ACAAAGACAAATACCATACAATTCCACTTATGAAGTATCTAAAGTAATCA
AAATCTTAGAAACAGAAAGTAGAATAGTGGTTACCAAGGCCGGGGGGGAA
GGAGGAAAAGGAGTTATTGTTCAAGAGTTTCAGTTTTACAAAATGATTCC
AGAGATCTGTTGCACAACAATGTGTATATACTGAACTGTACACTGAAAAA
TTGATAAGATGGTAAATAAAATACTATTAGTAAATGGGGCTAAGTTTTTT
TCTTTTTTTTTTTTCTTTTTTTTTTTTTTTTTGACAGAGTCTCACTCTGTTG
CCCAAGCTGGAGTGCAGTGGCACGATCTCGGTTCACTGTAACCTCTGCCT
CCTGGGTTCCAGCAATTCTCCTCCCTCAGCCTCCCGAGTAGCTGGGATTA
CAGGCACGCGCCACTACGCCCAGCTAATTTTTGTATTTTTAGCAGAGACA AluSz6
GTGTTTTGCCATGTTGGCCAGGCTTGTCTTGAACTCCTGACCTCAAGTGA
TCCGCCCACCTCGGCCTCCCAAAGGGCTGGGATTACAGGCATGAGCCACG
GCACCTAGCCCTAAAAAGTACTTTACCTTCAACCACAAGAAGCACCAGAA
AGGAGTTTAAAATTAGGGCATACTTTTACATTTTTGTATTGCAAAATATT
TGTATTATAAAATTCACAAGTGAATGTGTTTATATGCATGTACATATACA
TGCACACACAGTTCCCCCAACAGCTGGTAGGGAAGGGTCATCCTGGCCAG
AAGTGAACCTTCACACAGACCCTCCATTTGCTATGAATTGCTCACACTTG
GCAGGACCCAGGAAGACTGAATAATAAGACTAAAATTCCCAGCGATTAAT
ATTGAATCAGCACTTACGTAGTCTACCAGGCTCTTTAGATAACTTTTTCT
TTTTCAATTTTTTTTTTTTAATAGAGACAGGGTCTTACTATGTTGCCCAG
GCTGGTCTTGAACTCTTGAGCTCAAGCAATTACAGGCATGAGCCACTGTG
CCTGGGGCTTTTTGTTTTTGTTGTTTGTTTTTTGTTTTTGAGACTGAGTC
TCGCTCTGTAGCCCAGGTTGGAGTGCAGTGGTGCGATCTCGGCTGACTGC
AAGCTCCGCCTCCCGGGTTCACGCAATTCTCCTGCCTCAGTCTCCTGAGT
AGCTGGGATTACAGGTGCCCGCCACCAAGCCCAGCTAATTTTTTGTTTAT
TTTTAGTAGAGACGAGGTTTCACTATGTTGGCCAGACTGGCGTCTAACTC AluSx3
CTGACCTCGTGATCTGCCTGCCTCGGCCTCCCAAATTGCTGGGATTACAG
GCGTGAGCCACCGCGCCTGGCTGTTTTGTTTTGTTTTGTTTTTTGAGACA
GGGTTTCACTCCCGTCTCTCAGGCTGGAATGGAATGGTGTGCAATCTCGG
CTCACTGCAACCTCCACCTTCCAGGCTCAAGCAATTATCTTGCCTCGGCC
TCCCGAGTGGCTAGGAATACAGGCATGCGCCAGCACGCCTGGTTAATTTT
TGTAGAGACAAGGTTTTGCCGTGTTGCCCAGGCTGGTCTCAAACTCCTGA
ACTCAAGTGATCCTCCTGCCTCAGCCTCCCAAAGTGCTATAATTACAGGC

TTGAGCTACTGTGCCCAGCCTTCTTCTATTTTGAGACAGGGTCTCACTCT
GTCACCCAGGCTGGAATGCAGTGGCACGATCACCATTCACTGTAACCTCA
GCCTCCCAAGCTCAAGCAATCCTCCCATCTCAGCCTCCCAAGTAGCTGGG
ACCCACAGGCACACACCACCAGGCTTGACTAATTTTTTATATATTTTTGT
AGAGACAGGGTGTCCCATGTTGCCCAGCCTGGGAATTATTTAATTTAATC
CTTAACCATAAAAGGTAGGTAGCAGGACCCTGTTTGCCATGGCCTGAAAT
ATTCATCACATAGGACTCTCAGAGGCCACTTACCCAAGATCATAGACTCA
CTCAGCAGTACGGACCATGAATCCAGCTGTCTTTCATCCAGAACCTGAGG
GCAAAACAGTGGCCCCAGGGATGGCCAGTTGTCCAGGCCATGGGAGACCA
AAGGGATAGCACTTGAAGGAAGCTGGACTAGGGTAGGAACAGAAAGGGAA
TGACAGAAGACAGTCTCACCAGAGTGAACCTTAGGCCCATAAACCTGTCA
GAACCCATGATCAGGGCCCCTAACCCACCTTAGAGCCAGGGGAGGAAATA
AGAAGAGGGGACAGATGAACAGACCTCCTACTCAGGGACTTCAACGCAAG
GAGACCAGCTGCATTTATTGGAGACTTCCTCTAAGCTAGATTTCCTTTGT
AACAGCATTGTCAACCTTGTCAGGAAGAGATAGCTTGACCCATCTTCCAG
ATATGGGGAAAAAACTCAGATATGGAAACGATTTGCCTTAAGGGTACCCT
GCAAGTTGGAATTTGCTTCCAAGTTGTCTGGGCAAAAAATCCAGGCATCT
CCTTTCATGCCTCTCCTTGCCTCACATGCAAAACATGAGCAAGCTCTGCT
GGTTCTTCCTCTAAAATATAAGCCAAGTCTTTCTACTTATCCACGCTCCT
GCTACCCCAGCCCAAACTGCCATCATCACTCACCTGGACTTCATTCTAGA
AGCCTCCTCCCTGACCTCCCAGCTGCCCTGGAGAGTCCCTTCCAAACGCT
TCGGCTAGAAAGCTTTATTTTATTTATTTATTTATTTATTTATTTATTTA
GAGACAGAGTCTTACTCTGTCACCCAGGCTGGAGTGCAGTGGCACAATCT
CAGCCCACTGCAACCTCTGCCTCCCGGATTCAAGCAGTTCACCTGCCTCA
GCCTCCCAAGTAGCTGGAATTACAGGCACTTGCCACCATGCCCGGCTAAT
TTTTGTATTTTTAGTAAATACAAGGCTTCACCAT
