# Supplementary figures and images for: PPAR-Responsive Elements Enriched with Alu Repeats May Contribute to Distinctive PPARγ–DNMT1 Interactions in the Genome
Source: Cancers (Basel). 2021 Aug 7;13(16):3993. doi: 10.3390/cancers13163993 (PMC8391462; doi:10.3390/cancers13163993)

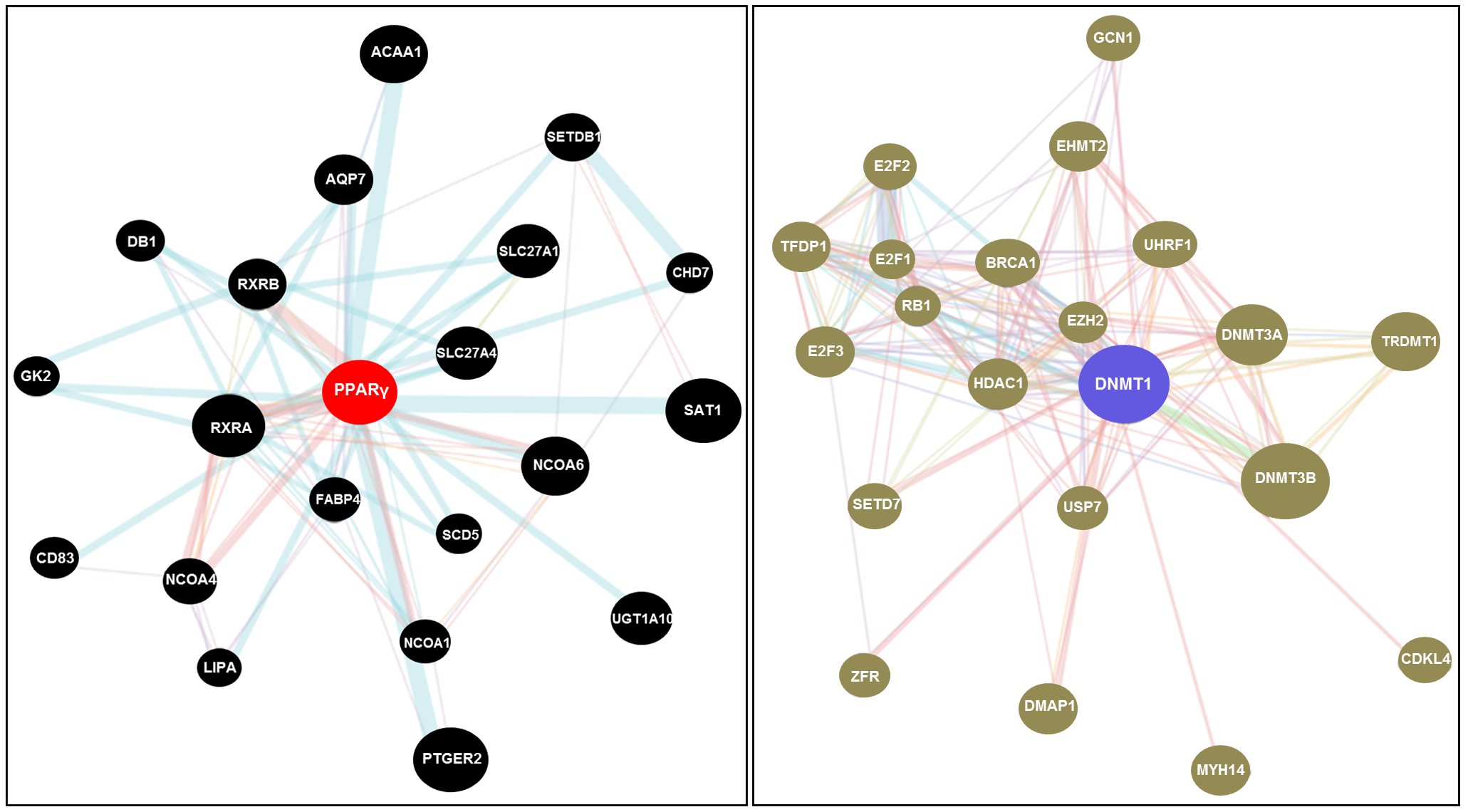

Supplement: Supplementary file 1 [file cancers-13-03993-s001.zip › Supplementary figure 1.png]

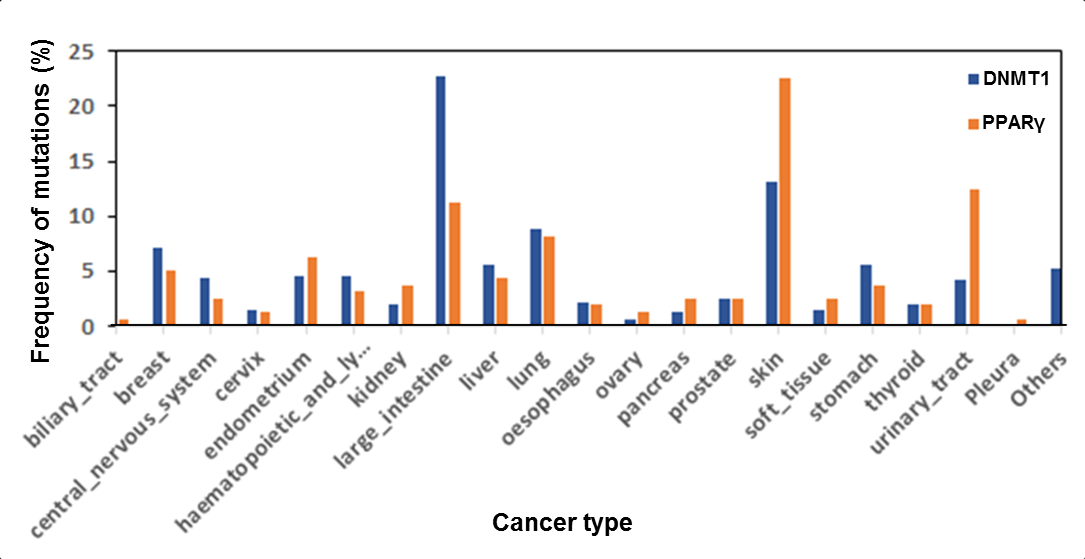

Supplement: Supplementary file 1 [file cancers-13-03993-s001.zip › Supplementary figure 2.png]

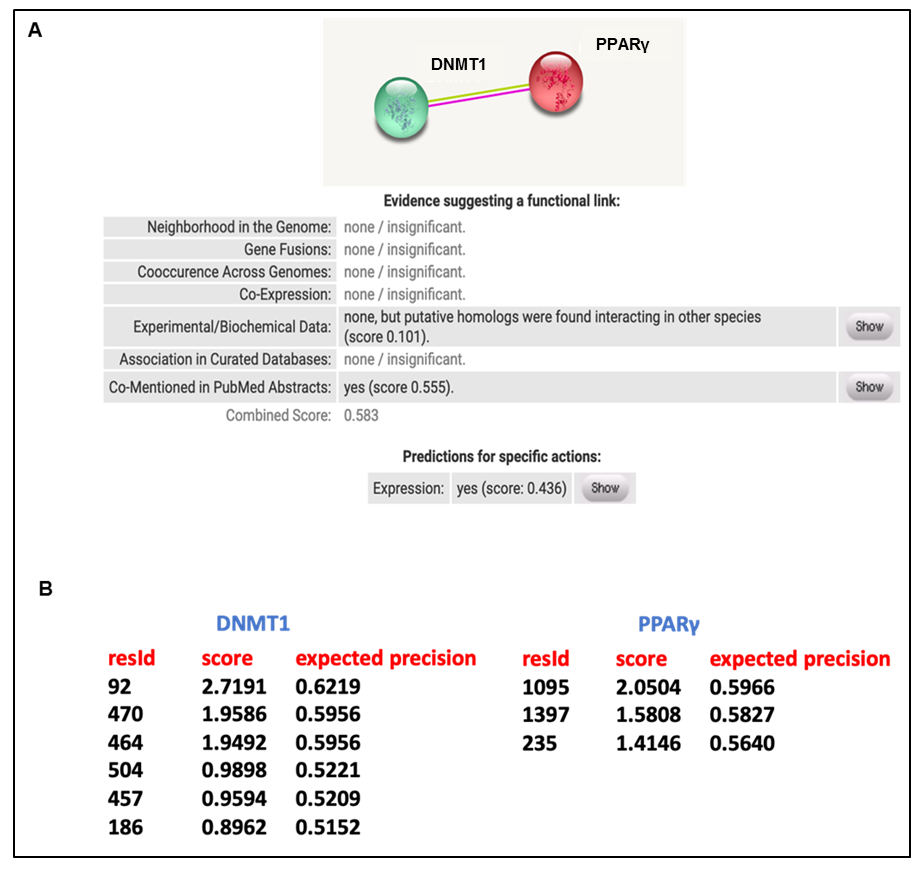

Supplement: Supplementary file 1 [file cancers-13-03993-s001.zip › Supplementary figure 3.png]
